# Biomechanical Investigation of Hallux Valgus Deformity Treated with Different Osteotomy Methods and Kirschner Wire Fixation Strategies Using the Finite Element Method

**DOI:** 10.3390/bioengineering10040499

**Published:** 2023-04-21

**Authors:** Kao-Shang Shih, Ching-Chi Hsu, Guan-Ting Huang

**Affiliations:** 1 Department of Orthopedic Surgery, Shin Kong Wu Ho-Su Memorial Hospital, Taipei 111, Taiwan; scorelin@gmail.com; 2 School of Medicine, College of Medicine, Fu Jen Catholic University, New Taipei City 242, Taiwan; 3 Department of Mechanical Engineering, National Taiwan University of Science and Technology, Taipei 106, Taiwan; m10603137@mail.ntust.edu.tw

**Keywords:** contact pressure, fixation stability, foot model, implant stress, metatarsal bone stress

## Abstract

The aim of this study was to propose a finite element method based numerical approach for evaluating various hallux valgus treatment strategies. We developed three-dimensional hallux valgus deformity models, with different metatarsal osteotomy methods and Kirschner wire fixation strategies, under two types of standing postures. Ten Kirschner wire fixations were analyzed and compared. The fixation stability, bone stress, implant stress, and contact pressure on the osteotomy surface were calculated as the biomechanical indexes. The results showed that the biomechanical indexes of the osteotomy and Kirschner wire fixations for hallux valgus deformity could be effectively analyzed and fairly evaluated. The distal metatarsal osteotomy method provided better biomechanical indexes compared to the proximal metatarsal osteotomy method. This study proposed a finite element method based numerical approach for evaluating various osteotomy and Kirschner wire fixations for hallux valgus deformity before surgery.

## 1. Introduction

Hallux valgus is the most common deformity of human forefoot. This foot deformity can lead to pain, redness, and swelling in the area of the first toe metatarsophalangeal joint [1,2]. Past research has reported that 23% to 35% of the general population have hallux valgus [3]. To relieve pain and correct the deformity, many osteotomy and fixation techniques are used for hallux valgus, such as open hallux valgus surgery, percutaneous hallux valgus surgery, and minimally invasive hallux valgus surgery [4,5,6,7]. In vivo and in vitro experiments have been applied to investigate the strengths and limitations of osteotomy and fixation techniques [8,9,10,11]. The main drawbacks of experimental approaches are experimental variability. The following parameters might affect variability: bone anatomy variation, bone density variation, a limited number of experimental subjects or specimens, and variation in the preparation of osteotomy and fixation techniques. Finite element method based numerical approaches have been widely used in orthopedic biomechanics [12,13,14]. This numerical approach could solve or reduce the drawbacks of experimental approaches. The finite element method has also been applied to investigate the problems of hallux valgus deformity. Zhang et al. [15] investigated the effects of distal chevron osteotomy displacement on metatarsal bone stress using the foot numerical model. Shih et al. [16] used a plate fixation to evaluate the effects of different hallux valgus treatments on the osteotomy fixation stability, implant stress, and bone stress, using both the single first metatarsal bone model and musculoskeletal lower extremity model. Brilakis et al. [17] investigated typical Mitchell’s procedure outcomes with or without bio-absorbable pins for hallux valgus using a three-dimensional finite element model of the foot. Xiang et al. [18] evaluated the longitudinal effects of a minimalist footwear running protocol for a mild/moderate hallux valgus patient using pre and post-intervention foot finite element models. Kirschner wire is a fixation technique for the treatment of hallux valgus deformity after performing metatarsal osteotomy [19,20,21]. There are many metatarsal osteotomy techniques and Kirschner wire fixation methods in clinical application. However, it is quite difficult to evaluate and compare the osteotomy techniques and fixation methods using in vivo or in vitro experiments due to the above-mentioned drawbacks. Thus, the purpose of this study was to investigate the biomechanical performance of the hallux valgus deformity with different metatarsal osteotomy methods and Kirschner wire fixation strategies using a finite element method based numerical approach.

## 2. Materials and Methods

### 2.1. Development of Hallux Valgus Deformity Model

**Figure 1:**
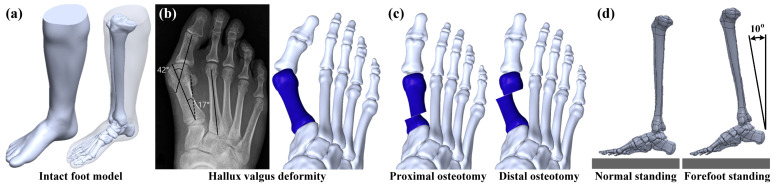
(**a**) The intact foot numerical model; (**b**) the foot model with hallux valgus deformity; (**c**) two types of metatarsal osteotomy methods; and (**d**) two types of foot standing postures.

A three-dimensional intact foot model was developed based on the Zygote Solid Skeleton model (Zygote Media Group, Inc., American Fork, UT, USA). This commercial foot model consists of the tibia, fibula, tarsals, metatarsals, and phalanges (Figure 1a). The bone parts of the foot were divided into cancellous bone and cortical bone using Geomagic Freeform and Geomagic Wrap (3D Systems, Rock Hill, SC, USA) and assembled using SolidWorks (Dassault Systèmes SolidWorks Corp, Waltham, MA, USA). The thickness for the cortical bone ranged from 1.5 to 3 mm. This thickness was referred to in a previous study [22]. A hallux valgus deformity model was developed according to a preoperative radiograph from a study by Lee et al. [23], which showed a 42° hallux valgus angle and a 17.4° intermetatarsal angle. The big toe of the foot was adjusted to mimic this hallux valgus deformity using SolidWorks (Figure 1b).

Hallux valgus deformity can be corrected with osteotomy and realignment [24,25]. In the present study, two types of metatarsal osteotomy methods were analyzed and investigated, including the proximal metatarsal osteotomy (PMO) and the distal metatarsal osteotomy (DMO). A PMO is defined as cutting the proximal region of the first metatarsal bone and realigning the position of the big toe. Similarly, a DMO is defined as cutting the distal region of the first metatarsal bone and realigning the position of the big toe (Figure 1c). Different foot postures can generate different foot loadings [26,27]. Thus, in the present study, two types of foot postures were analyzed and investigated, including the normal standing posture and the forefoot standing posture (Figure 1d). The normal standing posture mimicked the whole foot being on the ground, while the forefoot standing posture simulated only the forefoot on the ground. The inclined angle between the tibia shaft and the global vertical axis was 10° for the forefoot standing posture.

### 2.2. Various Osteotomy and Fixation Strategies

**Figure 2:**
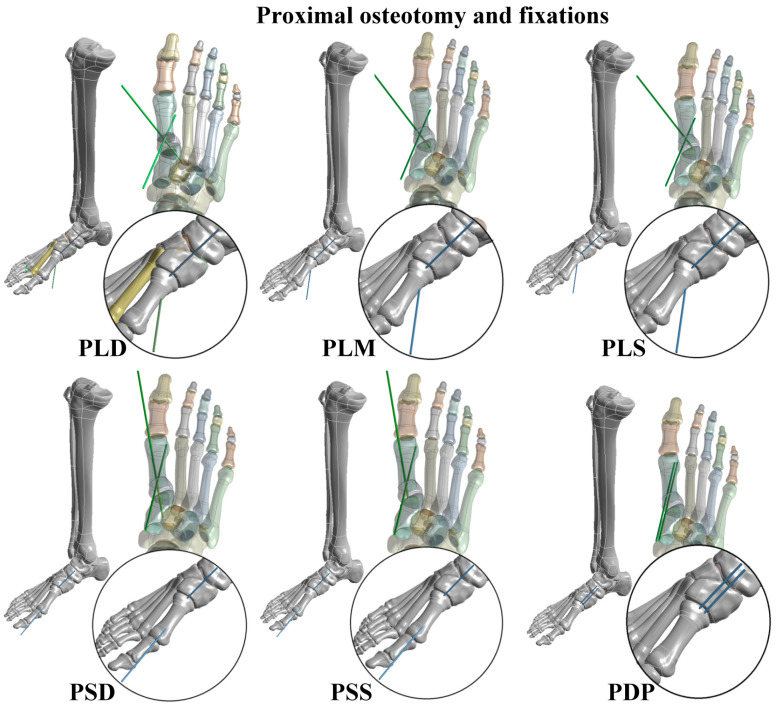
Six types of proximal metatarsal osteotomy (PMO) and Kirschner wire fixation strategies.

**Figure 3:**
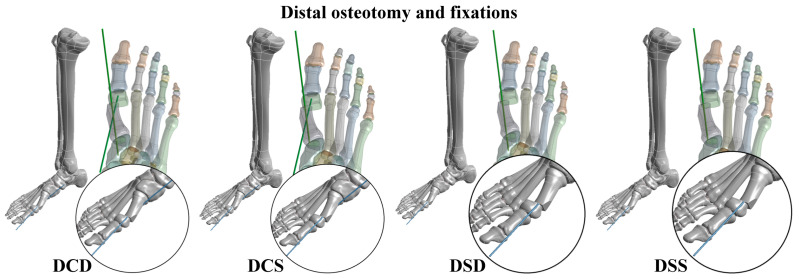
Four types of distal metatarsal osteotomy (DMO) and Kirschner wire fixation strategies.

Both the PMO and DMO were investigated in this study. Kirschner wire fixation was applied to fix the big toe after different metatarsal osteotomies. In the PMO, six types of Kirschner wire fixation methods were considered, including PMO combined with a large cross-angle and deep insertion fixation (PLD); PMO combined with a large cross-angle and medium insertion fixation (PLM); PMO combined with a large cross-angle and shallow insertion fixation (PLS); PMO combined with a small cross-angle and deep insertion fixation (PSD); PMO combined with a small cross-angle and shallow insertion fixation (PSS); and PMO combined with a double parallel wire fixation (PDP) (Figure 2). In the DMO, four types of Kirschner wire fixation methods were considered, including DMO combined with a cross-angle and deep insertion fixation (DCD); DMO combined with a cross-angle and shallow insertion fixation (DCS); DMO combined with a single and deep insertion fixation (DSD); and DMO combined with a single and shallow insertion fixation (DSS) (Figure 3). The Kirschner wires used in the present study had a diameter of 1.8 mm and their lengths were determined by each osteotomy and fixation strategy.

### 2.3. Finite Element Models

**Figure 4:**
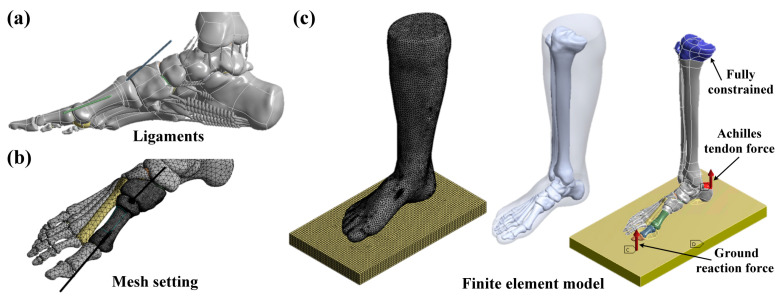
(**a**) The foot ligaments of the numerical model; (**b**) the mesh setting of the foot model with the osteotomy and Kirschner wire fixation; and (**c**) the finite element model of the foot with the loading and boundary conditions.

Three-dimensional finite element models of the foot with different osteotomy and fixation strategies were developed using ANSYS Workbench (ANSYS Inc., Canonsburg, PA, USA). The solid foot models were transferred into the finite element package via a Parasolid format. An isotropic linear elastic material was applied to the foot model. Sixteen types of foot ligaments were considered, including the anterior talofibular ligament; anterior tibiofibular ligament; calcaneofibular ligament; deep transverse metatarsal ligament; dorsal tarsometatarsal ligament; fibularis longus ligament; long plantar ligament; metatarsophalangeal joint capsules; phalangeal ligament; plantar calcaneonavicular ligament; plantar metatarsal ligament; posterior talofibular ligament; posterior tibiofibular ligament; superior fibular retinaculum; tibiocalcaneal ligament; and tibionavicular ligament (Figure 4a). All the foot ligaments were constructed using spring elements with tension-only behavior and defined with spring stiffness. This stiffness was calculated using the formula K=AE/L, where A is the cross-sectional area of the ligaments, E is the Young’s modulus of the ligaments, and L is the length of the ligaments. The input material properties of the foot models are listed in Table 1 [28,29]. Three-dimensional solid quadratic tetrahedral elements were used to discretize all parts of the foot (Figure 4b). To ensure precision of the numerical simulation [30], the meshing element size of the foot models was selected by conducting a convergence analysis. The convergence criterion was defined as the change in all output performances of less than 10% [31]. In the interface condition, a frictionless contact condition was applied between the Kirschner wires and bones using surface-to-surface contact elements (Contact 174 and Target 170). Additionally, the interface between the osteotomy faces of the metatarsal bone was also assumed to have frictionless contact. The remaining interfaces of the foot models were assumed to be bonded. In the boundary condition, all of the degrees of freedom were fixed at the proximal end of the tibia. In the loading condition, both an Achilles tendon force of 175 N and a ground reaction force of 350 N were considered. The Achilles tendon force was applied to the top surface of the calcaneus, and the ground reaction force was applied to the bottom surface of the ground part. These loadings were selected based on a study by Wong et al. [32] (Figure 4c). In the post-processing method, we calculated the maximum deformation of the first metatarsal bone; the maximum stress of the first metatarsal bone; the maximum stress of the Kirschner wires; and the average contact pressure of the first metatarsal bone. The maximum deformation of the first metatarsal bone was calculated to evaluate the stability of different osteotomy and fixation strategies. The maximum stress of the first metatarsal bone was calculated to evaluate the risk of bone fracture after surgery. The maximum stress of the Kirschner wires was calculated to evaluate the possibility of implant failure. Lastly, the average contact pressure of the first metatarsal bone was calculated to evaluate the bone healing rate and time after bone osteotomy. For the stress indexes, the von Mises criterion was used [33].

## 3. Results

### 3.1. Numerical Convergence

**Figure 5:**
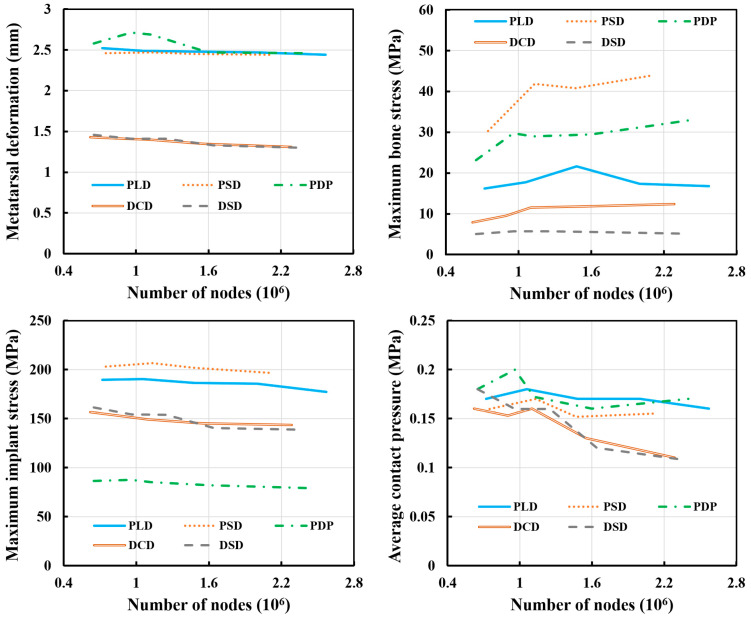
The results of the convergence study.

We conducted numerical convergence analysis of the hallux valgus deformity models after treatments. The element quality was used as the element quality criteria, with an average result of 0.8. We obtained the metatarsal deformation, maximum bone stress, maximum implant stress, and average contact pressure of the finite element models in terms of the number of nodes (Figure 5). The total number of nodes of the osteotomy and fixation methods for the convergence study ranged from 620,000 to 2,600,000. All performances of the finite element models converged properly when the number of nodes was larger than 2,000,000. The error due to the different number of nodes was less than 10%. All output performances that satisfied the convergence criterion were used in the following analyses and comparisons.

### 3.2. Fixation Stability

**Figure 6:**
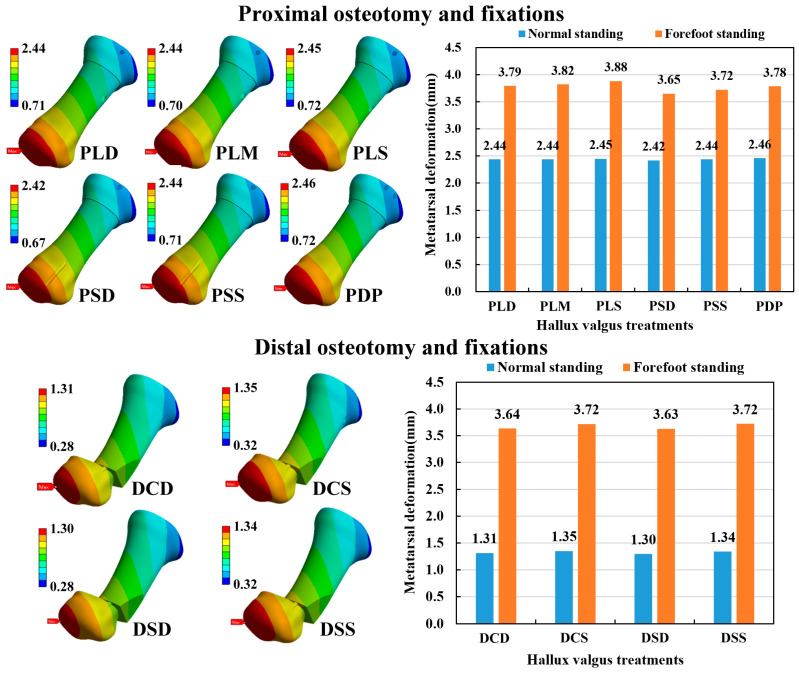
The displacement distribution of the numerical models and the results of the metatarsal deformation.

The maximum bone deformation was calculated to evaluate the stability of various osteotomy and fixation strategies. The maximum deformation occurred on the distal side of the bone for all osteotomy and fixation methods (Figure 6). In the case of proximal osteotomy and fixations (POFs), the metatarsal deformation of forefoot standing ranged from 3.65 to 3.88 mm, while normal standing ranged from 2.42 to 2.46 mm. Thus, the hallux valgus correction model with the forefoot standing posture had higher metatarsal deformation than the normal standing posture. However, different POF strategies had minor effects on metatarsal deformation under the same foot posture. In the case of distal osteotomy and fixations (DOFs), the metatarsal deformation of forefoot standing ranged from 3.63 to 3.72 mm, while normal standing ranged from 1.30 to 1.35 mm. Thus, the hallux valgus correction model with the forefoot standing posture had higher metatarsal deformation than the normal standing posture. Different DOF methods also had minor effects on metatarsal deformation under the same foot posture. Comparing the two kinds of osteotomy methods, the POF method revealed higher metatarsal deformation than the DOF method in the normal standing posture. However, the difference between the two kinds of osteotomy methods was insignificant in the forefoot standing posture (Figure 6).

### 3.3. Maximum Stress of First Metatarsal Bone

**Figure 7:**
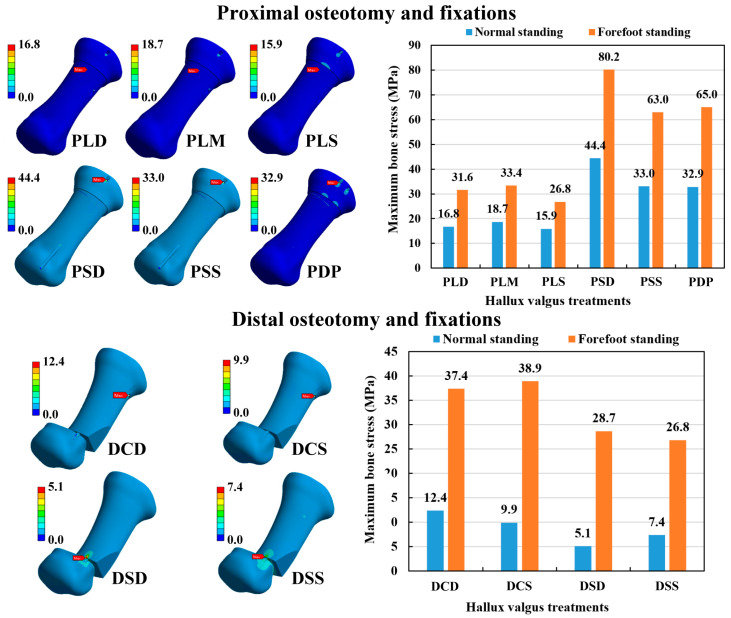
The bone stress distribution of the numerical models and the results of the maximum metatarsal bone stress.

The maximum bone stress was calculated to evaluate the risk of bone fracture after various osteotomy and fixation strategies. The maximum metatarsal bone stress occurred at the insertion hole of the Kirschner wires for all the osteotomy and fixation methods (Figure 7). In the POF conditions, the hallux valgus correction model with the normal standing posture had lower metatarsal bone stress than the forefoot standing posture. The bone stress of the large cross-angle fixations (PLD, PLM, and PLS) ranged from 15.9 to 33.4 MPa, while the bone stress of the other fixations ranged from 33.0 to 80.2 MPa. Thus, POM combined with large cross-angle fixations (PLD, PLM, and PLS) had lower bone stress than small cross-angle fixations (PSD and PSS) and double parallel wire fixation (PDP) under the same foot posture. Under the DOF conditions, the hallux valgus correction model with the normal standing posture had lower metatarsal bone stress than with the forefoot standing posture. The bone stress of single Kirschner wire fixations (DSD and DSS) ranged from 5.1 to 28.7 MPa, while the bone stress of the other fixations ranged from 9.9 to 38.9 MPa. Thus, DMO combined with single Kirschner wire fixations (DSD and DSS) had lower metatarsal bone stress than with two cross-angle Kirschner wire fixations (DCD and DCS) under the same foot posture. Comparing the two kinds of osteotomy methods, the DOF method revealed relatively lower metatarsal bone stress than the POF method (Figure 7).

### 3.4. Maximum Stress of Fixation Implant

**Figure 8:**
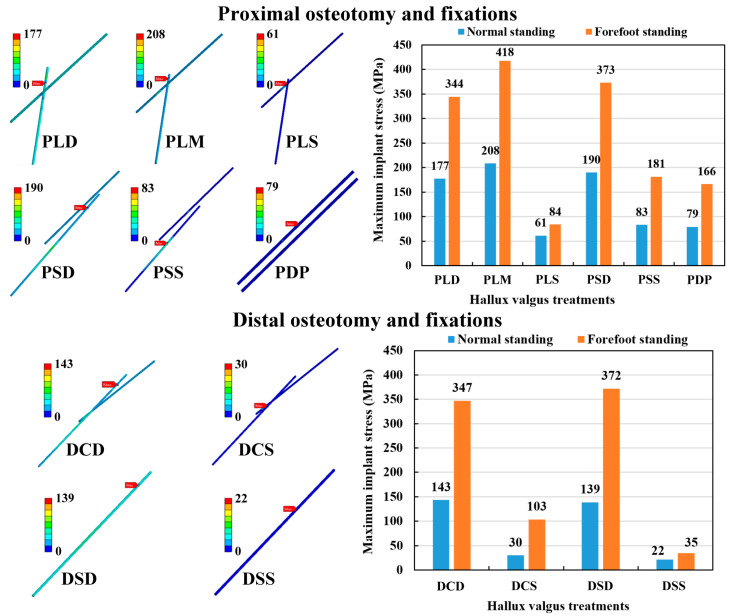
The implant stress distribution of the numerical models and the results of the maximum Kirschner wire stress.

The maximum implant stress was calculated to evaluate the possibility of the Kirschner wire failure. The maximum implant stress occurred mainly on the wire’s exterior surface near the osteotomy site for all the osteotomy and fixation strategies, except for the PSS fixation strategy, in which it occurred at the metatarsophalangeal joint; and in the DCD, DSD, and DSS strategies, in which it occurred at the end of the metatarsal bone (Figure 8). In the POF conditions, the implant stress of forefoot standing ranged from 84 to 418 MPa, while normal standing ranged from 61 to 208 MPa. Thus, the hallux valgus correction model with the normal standing posture had lower implant stress than the forefoot standing posture. The PMO combined with either shallow insertion fixations (PLS and PSS) or double parallel wire fixation (PDP) had lower implant stress than the other fixations under the same foot posture. In the DOF conditions, the implant stress of forefoot standing ranged from 35 to 372 MPa, while normal standing ranged from 22 to 139 MPa. Thus, the hallux valgus correction model with the normal standing posture had lower implant stress than the forefoot standing posture. The DMO combined with shallow insertion fixations (DCS and DSS) had lower implant stress than the other fixations under the same foot posture. Comparing the two kinds of osteotomy methods, the DOF method revealed slightly lower implant stress than the POF method (Figure 8).

### 3.5. Average Contact Pressure on Osteotomy Surface

**Figure 9:**
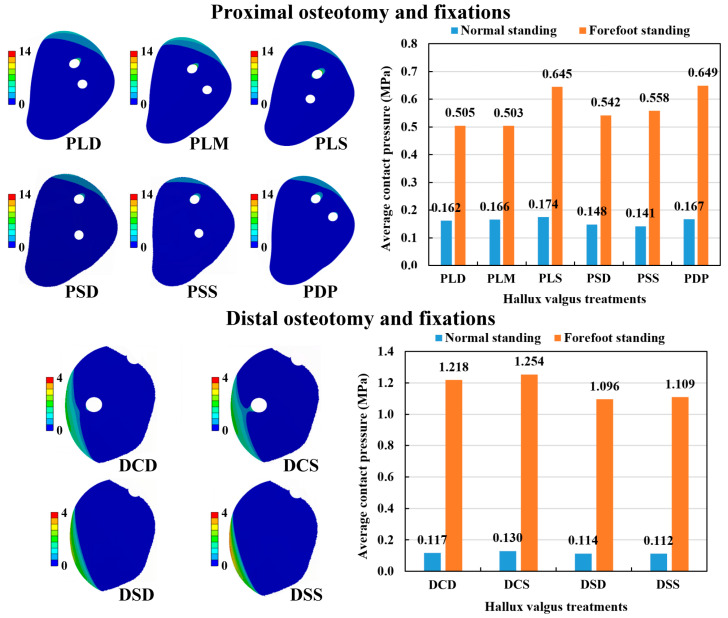
The contact pressure distribution on the osteotomy surface of the numerical models and the results of the average contact pressure.

The contact pressure distribution on the osteotomy surface of various fixation methods was obtained (Figure 9). Based on these contact pressure results, we calculated the average contact pressure, which summarized the contact pressure from the osteotomy surface divided by the surface area. In the POF conditions, the average contact pressure of forefoot standing ranged from 0.503 to 0.649 MPa, while normal standing ranged from 0.141 to 0.174 MPa. Thus, the hallux valgus correction model with the forefoot standing posture had higher average contact pressure than the normal standing posture. The PMO combined with either large cross-angle and shallow insertion fixation (PLS) or double parallel wire fixation (PDP) had higher average contact pressures than the other fixations under the same foot posture. In the DOF conditions, the average contact pressure of forefoot standing ranged from 1.096 to 1.254 MPa, while normal standing ranged from 0.112 to 0.130 MPa. Thus, the average contact pressure of the hallux valgus correction model with the forefoot standing posture was significantly higher than the normal standing posture. The DMO combined with either of two cross-angle Kirschner wire fixations (DCD and DCS) had higher average contact pressures than single Kirschner wire fixations (DSD and DSS) under the same foot posture. Comparing the two kinds of osteotomy methods, the POF method revealed higher average contact pressure than the DOF method in the normal standing posture. However, the latter revealed higher average contact pressure than the former in the forefoot standing posture (Figure 9).

## 4. Discussion

Numerous osteotomy and fixation techniques for hallux valgus deformity have been proposed [34]. However, the best or most suitable techniques vary due to the surgeon’s choice of surgical approach [35]. Proposing a scientific approach that can evaluate numerous osteotomy and fixation techniques before clinical surgery is an important issue. It is known that the numerical approach has the advantage of exploring ‘what if’ questions without having to experiment [36]. In the present study, a finite element method based numerical approach was proposed as a possible scientific approach for evaluating the strengths and limitations of osteotomy and fixation techniques. The effects of various osteotomy methods and fixation strategies on the predicted biomechanical outcomes of the hallux valgus treatments can be effectively analyzed and evaluated using finite element models. Compared to experimental approaches, numerical models can predict first metatarsal bone stress, Kirschner wire stress, and average contact pressure on the osteotomy surface. This is the main benefit of the numerical approach, since these stress and pressure outcomes are quite difficult to measure using experimental approaches.

The PMO with various Kirschner wire fixations was analyzed in the present study. Four different biomechanical indexes of the hallux valgus treatments were calculated, including fixation stability, metatarsal bone stress, Kirschner wire stress, and average contact pressure on the osteotomy surface. Different fixation strategies have no significant differences on fixation stability. However, metatarsal bone stress, Kirschner wire stress, and average contact pressure varied when different Kirschner wire fixations were applied. Kirschner wire fixations with the use of a large cross-angle (PLD, PLM, and PLS) can reduce metatarsal bone stress. Additionally, Kirschner wire fixations with the use of either a large cross-angle and shallow insertion (PLS) or the double parallel wire insertion (PDP) can reduce Kirschner wire stress and increase average contact pressure. In summary, the PMO combined with a large cross-angle and shallow insertion fixation (PLS) reveals better biomechanical indexes compared to other fixations. Meanwhile, the DMO with various Kirschner wire fixations was also evaluated in this study. The various Kirschner wire fixations revealed minor differences in fixation stability and average contact pressure. For metatarsal bone stress and Kirschner wire stress, the DMO combined with single Kirschner wire fixations (DSD and DSS) revealed lower metatarsal bone stress, while the DMO combined with shallow insertion fixations (DCS and DSS) showed lower Kirschner wire stress. We conclude that the DMO combined with a single and shallow insertion fixation (DSS) reveals better biomechanical indexes compared to other fixations.

Comparing two different metatarsal osteotomy methods, the DMO method had better fixation stability, lower metatarsal bone stress, and lower Kirschner wire stress than the PMO method for both the normal standing and forefoot standing postures. However, these two osteotomy methods had diverse results in terms of average contact pressure. The PMO method revealed higher average contact pressure in the normal standing posture, but the DMO method showed higher average contact pressure in the forefoot standing posture. Thus, we suggest the DMO method for the treatment of hallux valgus deformity. This finding verifies a clinical study conducted by Lee et al. [37]. They suggested that distal osteotomy with a distal soft-tissue procedure is an effective and reliable method for correcting moderate to severe hallux valgus compared to proximal osteotomy with a distal soft-tissue release. Although these two studies had similar findings, there is still a question of which metatarsal osteotomy method is best to treat hallux valgus deformity. Trnka [38] concluded that a variety of metatarsal osteotomies have been described but many of these osteotomies have been abandoned over the years. The main reason was that one single procedure is not capable of correcting all types of hallux valgus deformities. Thus, developing a quick and non-invasive evaluation method such as the finite element method is worthy and necessary [39].

Stress is a sensitive index, and it is susceptible to local geometry. In the present study, the insertion hole of the Kirschner wires created this local geometry. Thus, the maximum metatarsal bone stress occurred at the insertion hole of the Kirschner wires for all the osteotomy and fixation methods. Some treatment strategies revealed implant stresses that are higher than material yield strength. This is due to the assumption of a linear elastic and isotropic material model. Although some treatment strategies revealed implant stresses that are higher than material yield strength, we still can find relatively good treatment strategies where the implant stresses are lower than the material yield strength. In addition, contact pressure was relatively low when the Kirschner wire fixation provided more rigid fixation. Based on the contact pressure distribution, all the Kirschner wire fixation strategies provided stronger fixation. Thus, the contact pressure was lower around the Kirschner wire holes. In the present study, the average contact pressure on the osteotomy surface was calculated to quantify the results of different treatment strategies. The treatment strategy with higher average contact pressure was considered to have better bone healing rate. Selection of stress criterion should be paid more attention in order to avoid erroneous conclusions. The von Mises stress represents an equivalent stress that considers all normal stresses and shear stresses. In the present study, an isotropic linear elastic material was applied to model the hard bone tissues and fixation implants. Thus, this stress criterion is a suitable criterion.

Foot motion, muscle performance, and posture are other parameters that can affect the biomechanics of hallux valgus deformity treatment [40,41]. To discover the potential effects of foot parameters on the biomechanics of hallux valgus deformity treatment, foot models with either the normal standing posture or the forefoot standing posture were considered. Foot models with the forefoot standing posture had larger metatarsal deformation, higher metatarsal bone stress, higher Kirschner wire stress, and higher average contact pressure than the normal standing posture. The normal standing posture considers the contact between the whole foot and the ground, so the ground reaction force could be shared through the whole foot. However, the forefoot standing posture only considers the contact between the forefoot and the ground. This is the reason why the forefoot standing posture resulted in larger or higher biomechanical indexes compared to the normal standing posture. This finding verifies Guiotto et al.’s study [42]. They found that the peak plantar pressure on the forefoot in the push-off posture is higher than in the midstance posture. The higher peak plantar pressure on the forefoot represents more loading that the foot typically takes.

The present study has some potential limitations. First, an isotropic linear elastic material was used to model the hard tissues of the foot. Additionally, all the foot ligaments were modeled as linear and tension-only spring elements. The material failure or nonlinear behavior of the hard and soft tissues cannot be investigated via the numerical approach proposed by the present study. The numerical model could only provide relative comparisons and not absolute differences. This limitation could be improved using orthotropic or anisotropic material models. Second, there are a lot of metatarsal osteotomy methods that have been used for hallux valgus treatment. In the present study, a perfect flat osteotomy cut was applied to both the PMO method and the DMO method. The effects of various osteotomy cuts on the biomechanical indexes have not been investigated. Third, both the normal standing posture and the forefoot standing posture were considered to investigate their effects on the biomechanical indexes. These foot postures were extracted from a gait cycle. The effects of the full gait cycle on the biomechanical indexes of the hallux valgus treatment need further investigation. Fourth, first ray hypermobility is one of the predisposing factors for the development of hallux valgus [43]. This factor was not considered in the present study. Finally, due to the model complexity and computational efficiency, a variation of 10% was used as the convergence criterion in the present study. A tighter convergence criterion can be considered for further investigation.

## 5. Conclusions

The hallux valgus deformity with various osteotomy and Kirschner wire fixation techniques were effectively analyzed and fairly evaluated using a finite element method based numerical approach. The PMO combined with a large cross-angle and shallow insertion fixation (PLS), as well as the DMO combined with a single and shallow insertion fixation (DSS), revealed better biomechanical indexes among various POF and DOF strategies. The DMO method provides better fixation stability, lower metatarsal bone stress, and lower Kirschner wire stress and is suggested for the treatment of hallux valgus deformity over the PMO method. Furthermore, the efficient numerical evaluation technique proposed by this study could provide useful information to surgeons before performing hallux valgus deformity surgery.

## Figures and Tables

**Table 1:** The material properties of the numerical models for the treatment of hallux valgus deformity.

Materials	Young’s Modulus (MPa)	Poisson’s Ratio	Stiffness (N/mm)
Articular cartilage	3.4	0.40	-
Cancellous bone	100	0.30	-
Cortical bone	10,000	0.34	-
Kirschner wires (stainless steel)	200,000	0.30	-
Soft tissue	1.15	0.49	-
Anterior talofibular ligament	-	-	47.3
Anterior tibiofibular ligament	-	-	115.9
Calcaneofibular ligament	-	-	24.9
Deep transverse metatarsal ligament	-	-	78
Dorsal tarsometatarsal ligament	-	-	115.2
Fibularis longus ligament	-	-	67.2
Long plantar ligament	-	-	28.1
Metatarsophalangeal joint capsules	-	-	136.5
Phalangeal ligament	-	-	169.1
Plantar calcaneonavicular ligament	-	-	65.7
Plantar metatarsal ligament	-	-	15.7
Posterior talofibular ligament	-	-	25.5
Posterior tibiofibular ligament	-	-	257.8
Superior fibular retinaculum	-	-	25.6
Tibiocalcaneal ligament	-	-	109.5
Tibionavicular ligament	-	-	14.5

## Data Availability

The data presented in this study are available on request from the corresponding author.
